# Characterization of DnaB–DnaG Interaction in *M. tuberculosis* Using Small‐Angle X‐ray Scattering‐Based Dissociation Assay

**DOI:** 10.1002/cbic.202500289

**Published:** 2025-07-01

**Authors:** Dayan A, Ilic S, Akabayov B

**Affiliations:** ^1^ Department of Chemistry Ben‐Gurion University of the Negev Beer‐Sheva 8410501 Israel

**Keywords:** DnaB, DnaG, protein–protein interactions, small‐angle X‐ray scattering dissociation assay

## Abstract

The complex interactions between helicase and primase, two key components of the replisome involved in DNA replication in *Mycobacterium tuberculosis* are studied. Utilizing purified, complementary domains of these proteins, a surface plasmon resonance (SPR) analysis and a cross‐linking assay to characterize their binding dynamics are employed. The SPR analysis reveals a binding dissociation constant of 0.21 ± 0.08 μM, and the cross‐linking assay suggests the possible formation of a heterodimer species. Importantly, a small‐angle X‐ray scattering dissociation assay to study the dynamic interactions between the proteins in solution is utilized. The findings provide new opportunities for targeted therapeutic strategies aimed at DNA replication in *M. tuberculosis* by revealing the structural interplay between helicase and primase.

## Graphical abstract

The study characterizes interactions between DnaB helicase and DnaG primase in *Mycobacterium tuberculosis* using small‐angle X‐ray scattering, surface plasmon resonance, and cross‐linking. The findings reveal that DnaG forms dimers in solution, which are destabilized upon DnaB binding. This interaction suggests a regulatory mechanism in DNA replication as well as potential targets for antibacterial drug development.

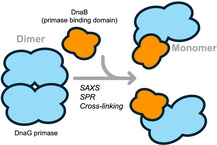

## 1 Introduction

**Figure 1:**
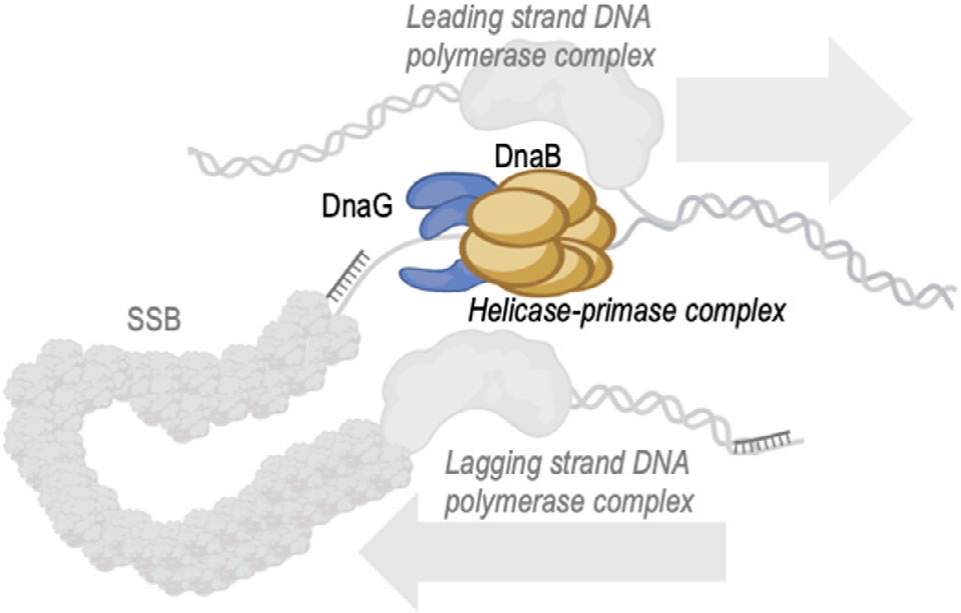
Assembly of proteins at the DNA replication fork of *M. tuberculosis*. DnaB helicase and DnaG primase are recruited along with DNA pol III holoenzyme and together commence the bidirectional synthesis of a new DNA molecule. A helicase ring (gray) unwinds double‐stranded DNA to expose the two individual strands. One strand is copied continuously (leading), and the other, discontinuously (lagging) by two DNA polymerase complexes (light gray). DNA polymerases use short RNA primers synthesized by DnaG primase (green) on the lagging strand to form Okazaki fragments. The RNA primers on the lagging strand are degraded, and the gaps are filled and ligated by further enzymatic activities in the replication fork to form a single continuous DNA strand.^[^ ^43^ ^]^ The illustration was created based on the crystal structure of bacteriophage T7 replisome^[^ ^44^ ^]^ PDBID 5IKN using PyMol (https://www.pymol.org).

Analyzing the formation of protein complexes poses significant challenges, especially when the binding affinity between the proteins is low or when the protein complex is formed transiently as a stage in a biochemical pathway within a cell.^[^ ^1^ ^]^ One such pathway is the process of DNA replication, which is orchestrated by the replisome^[^ ^2^ ^]^ (**Figure** **1**), a substantial molecular complex comprised of proteins that synthesize new DNA strands from an existing DNA molecule. In this process, protein interactions at the DNA replication fork are vital for maintaining accurate DNA duplication,^[^ ^3^ ^]^ and crucial among these interactions is the one between DnaB helicase and DNA primase (DnaG).^[^ ^4^, ^5^ ^]^ DnaB helicase separates dsDNA into two strands in an ATP‐dependent manner.^[^ ^6^ ^]^ During DNA replication, DnaB becomes transiently associated with DNA primase (DnaG), which catalyzes the formation of RNA primers for the synthesis of Okazaki fragments.^[^ ^7^ ^]^ In bacteria, primase binding to helicase enhances both the primase's specificity and the helicase's dsDNA unwinding activity.^[^ ^8^, ^9^, ^10^ ^]^ This cooperative interaction helps to coordinate the unwinding of the DNA helix with the synthesis of the new DNA strands, ensuring the accuracy of the replication process and starting the replication fork “clock”.^[^ ^11^ ^]^ The binding affinity between helicase and primase varies among species, and the exact compositions of helicase and primase subunits within an active primosome are still unknown.^[^ ^12^ ^]^

Structurally, DnaB helicase is a hexamer that is made up of identical subunits and comprises two domains—an N‐terminal domain that is responsible for protein–protein interactions and a C‐terminal catalytic domain with a RecA‐like core fold.^[^ ^4^, ^13^ ^]^ DnaG primases consist of a zinc‐binding domain (ZBD), an RNA polymerase domain (RPD), and a C‐terminal helicase‐binding domain (HBD).^[^ ^14^ ^]^ The available solved structures of DnaG include those for RPD and HBD,^[^ ^14^ ^]^ two of its three structural domains. The nature of the interaction between DnaB and DnaG and the complex structure they form have been the subject of several studies.^[^ ^12^, ^14^, ^15^, ^16^, ^17^ ^]^ However, the complex between the helicase and the primase exhibits a highly dynamic structure, forming transiently during the turnover steps of DNA replication.^[^ ^10^ ^]^ Such transient binding between proteins and the temporary formation of larger complex species complicates the ability to monitor and characterize—both structurally or functionally—these fleeting structures by using conventional structural or biochemical methodologies. Nonetheless, addressing the stabilization of nonstable protein species has been the focus of various strategies, including the utilization of nonhydrolyzable nucleoside‐tri‐phosphates,^[^ ^18^ ^]^ noncleavable substrates,^[^ ^19^ ^]^ and modified protein residues.^[^ ^20^ ^]^ These methods aim to retain a stable, albeit static, conformation of the relevant complex. However, while these approaches can extract some structural information, they often provide only partial insights and they can even occasionally obscure crucial data about the active species.

Similarly, traditional techniques for examining static structures, such as X‐ray crystallography or cryo‐electron microscopy, can offer valuable structural and physical insights into the nature of inter‐species binding, but they provide a limited perspective at best and often completely fail to describe dynamic processes effectively. Nuclear magnetic resonance (NMR), in contrast, provides dynamic descriptions, although the size of macromolecules and sample preparation can be limiting. The solution to these limitations lies in a combination of small‐angle X‐ray scattering (SAXS) with protein reconstitution assays, which provides insight into structural events between binding species in solution.^[^ ^21^ ^]^ While glutaraldehyde cross‐linking does create nonspecific interactions by targeting surface lysines, it effectively complements SAXS because it stabilizes transient protein–protein interactions. The design of these assays offers two particular advantages: they detect weak protein–protein interactions with dissociation constants (*K*_D_) in the sub‐millimolar range, and they do not manipulate the components in ways that would alter their binding affinity,^[^ ^21^, ^22^, ^23^ ^]^ namely, the dissociation constant is not physically changed by altering the binding species. Instead, increasing the amount of one component while maintaining a constant amount of the other “pushes” complex formation along the titration, contingent on the dissociation constant. For instance, if the *K*_D_ between components A and B is 100 μM, a sample containing 20 times more component A than component B would be needed to maintain over 90% of the A + B complex.^[^ ^24^ ^]^ In addition to the two particular advantages mentioned above, SAXS reconstitution assays offer other advantages over traditional structural methods, even though their resolution is lower: They are quick, cost‐effective, easy to analyze, and require only a small amount of protein samples.

## 2 Results and Discussion

To date, *Escherichia coli* has served as the primary model for studying bacterial DNA replication, providing insights into the structure and function of the DNA replication apparatus. However, it has been suggested that DNA replication features in *E. coli* do not necessarily represent those in other bacteria.^[^ ^25^ ^]^ For example, *Mycobacterium tuberculosis* has a distinct mechanism for DNA replication,^[^ ^26^ ^]^ involving multiple, complex interactions, such as that between DnaB helicase and DnaG primase. In this organism, the minimal replisome arrangement in *M. tuberculosis* required for coordinated DNA synthesis remains largely unknown, as do the roles of many protein components at the replication fork.^[^ ^27^ ^]^ Our study uses complementary interacting domains to examine the binding dynamics between DnaB helicase and DnaG primase of *M. tuberculosis*. We obtained the interacting domains of each protein through overexpression and purification (see Section 4). The inclusion of T7 primase as a model system reflects its structural similarity to bacterial primases and the availability of detailed crystal structures. However, comparisons with *M. tuberculosis* systems remain the study's primary focus.

### 2.1 DnaG Primase Forms Stable Dimers in Solution

Proteins involved in DNA replication form large groups to enhance interactions with DNA, increase binding specificity, stabilize protein domains, and regulate enzymatic activity. While DnaB forms a ring structure of protein subunits that encircles the DNA, the oligomeric state of DnaG is unknown. The crystal structures of *E. coli* DnaG primase (PDB ID: 3B39^[^ ^28^ ^]^) and *Helicobacter pylori* (PDB ID: 4EHS^[^ ^15^ ^]^) indicate a homodimeric arrangement of DnaG, with the two primase domains facing opposite directions. However, this homodimeric DnaG arrangement could result from crystal packing and not necessarily the native arrangement. It is, therefore, essential to establish stable primase dimers in the test tube before introducing helicase, as this likely represents the active state of primase in solution. To elucidate the oligomeric state of primase in solution, we utilized the primase domain of phage T7 gene 4 protein (T7 primase), which has been widely recognized as a structural model for DnaG primase. In addition to the structural conservation within the DnaG‐like prokaryotic primase family, other properties, such as the low molecular weight, easy production process, stability in high concentrations, and the available crystal structure (PDB ID: 1NUI^[^ ^29^ ^]^) make T7 primase an ideal candidate for NMR‐based structural analysis.^[^ ^29^ ^]^ The addition of 4‐mer RNA likely stabilizes dimer formation by bridging transient protein–protein interaction surfaces, as observed in other homologous systems.

**Figure 2:**
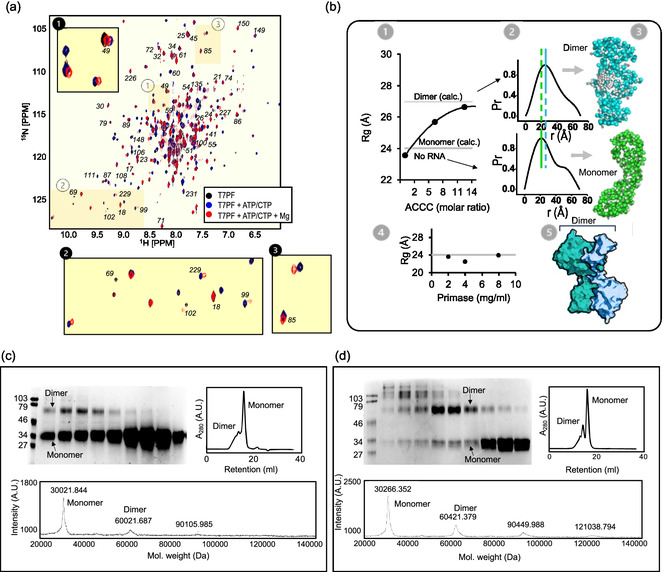
Dimerization of T7 DNA primase. a) [^1^H, ^15^N]‐TROSY HSQC spectrum of ^15^N‐deuterated T7 primase; black—T7 primase alone, blue—T7 primase with 1 mM ATP/CTP, and red—T7 primase with 1 mM ATP/CTP and 10 mM MgCl_2_. Insets show magnified views of specific areas (labeled 1, 2, 3), indicating changes in chemical shifts upon the addition of ATP/CTP/MgCl_2_. Assignments of the observed resonances were derived from published NMR assignments of T7 primase.^[^ ^30^ ^]^ The original assignment map, which accounts for 70% of the resonances, is shown in Figure S1a, Supporting Information. b) SAXS structural analysis and visualization of T7 primase. b1) Radius of gyration (*R*_g_) of T7 primase (50 μM) in the presence of increasing amounts of 4‐mer RNA primers (5′‐ACCC‐3′). The gray lines represent theoretical *R*_g_ values for the T7 DNA primase monomer (*R*_g_ = 24 Å) and dimer (*R*_g_ = 27 Å) calculated using the crystal structure of T7 primase (PDBID: NUI^[^ ^29^ ^]^), as a reference structure, and CRYSOL software.^[^ ^31^ ^]^ Raw SAXS profiles are presented in Figure S2a, Supporting Information. b2) Distance distribution functions of the T7 primase alone (lower portion) and T7 primase in the presence of RNA (upper portion), obtained using the computer program GNOM.^[^ ^38^ ^]^ b3) The corresponding ab initio SAXS model of gp5 obtained using the ab‐initio modeling program GASBOR^[^ ^45^ ^]^ that uses SAXS data to reconstruct a protein structure by representing it as an assembly of dummy beads. b4) *R*_g_ determined using an in‐house script for T7 primase at three concentrations (2, 4, and 8 mg mL^−1^). Raw SAXS profiles are presented in Figure S2b, Supporting Information. b5) Crystal structure indicates plausible dimerization of T7 primase (PDBID: NUI^[^ ^29^ ^]^). c) Fe(II)‐catalyzed cross‐linking of T7 primase. d) Cross‐linking of T7 primase with glutaraldehyde. The reaction products were separated by size‐exclusion chromatography (top right), and the fractions were analyzed using SDS‐PAGE (top left). One fraction, corresponding to each cross‐linking reaction, was selected, based on the highest dimer‐to‐monomer ratio, and analyzed by MALDI‐TOF (bottom panel).

We conducted a thorough biochemical and structural investigation using NMR and SAXS alongside various cross‐linking techniques. Protein samples for these experiments were prepared in the presence of ATP/CTP, RNA, and magnesium to stabilize the proteins in their active state. The [^15^N, ^1^H]‐TROSY‐heteronuclear single quantum coherence (HSQC) NMR spectra showed changes in the chemical environment of the surface amino acids upon the addition of magnesium and ATP/CTP, and the NMR spectral peaks were significantly less dispersed, indicating the formation of a larger, presumably dimeric, structure (**Figure** **2**a). The residue‐specific chemical shift perturbations observed in the [^1^H, ^15^N]‐TROSY HSQC spectra upon addition of ATP/CTP and MgCl_2_ (Figure 2a) correspond primarily to surface residues of the T7 primase structure. The assignment of these resonances to specific amino acid residues has been previously published,^[^ ^30^ ^]^ where extensive triple‐resonance experiments established 70% of the backbone resonance assignments, enabling identification of residues involved in the formation of the dimeric primase structure (Figure S1a–c, Supporting Information). This interpretation is based on prior evidence from both earlier and recent studies. In the foundational NMR study by Kato et al.,^[^ ^29^ ^]^ T7 primase displayed a distinct pattern of overlapped peaks under catalytically active conditions, suggesting that the protein adopts a higher‐order structural state with reduced tumbling and slower dynamics, which suggests increased molecular weight. In contrast, when measured in the absence of these cofactors, the spectra showed well‐dispersed peaks with minimal overlap, indicative of a monomeric 27 kDa species {Ilic, 2016 #489}. Building upon these observations, we hypothesized that ATP/CTP and Mg^2^^+^, which are required to initiate primer synthesis, stabilize a catalytically competent dimeric form of T7 primase.

To corroborate the NMR results, we employed a SAXS assay in the presence of ATP/CTP, RNA, and Mg^2+^, similar to the NMR studies.

**Figure 3:**
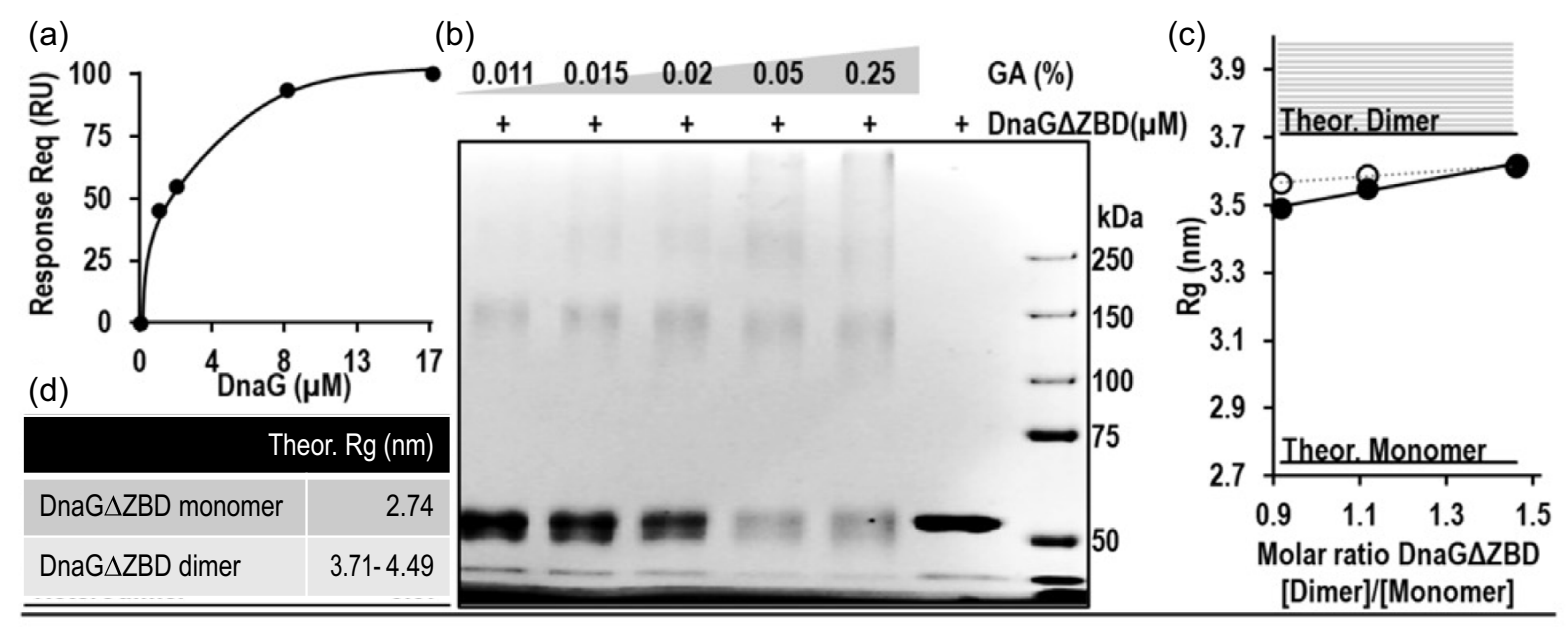
Dimerization of DnaG from *M. tuberculosis*. a) SPR analysis of soluble full‐length DnaG binding to the full‐length DnaG immobilized on a GLC sensor chip by amino coupling. The signal observed at equilibrium was plotted as a function of soluble DnaG concentration (raw data are available in Figure S1c, Supporting Information). b) Formation of DnaGΔZBD dimers after crosslinking with increasing concentrations of glutaraldehyde observed by SDS‐PAGE. Gel bands around 60 and 120 kDa correspond to DnaGΔZBD monomers and dimers, respectively. c) Values of *R*_g_ obtained by SAXS of monomeric and dimeric state of DnaGΔZBD at concentrations of 5, 7, and 11 μM. Solid black circles represent the calculated *R*_g_ values of DnaGΔZBD in solution, and empty circles represent experimental *R*_g_ values. The raw SAXS profiles, along with their corresponding Guinier plots, are shown in Figure S1d, Supporting Information. d) Theoretical *R*_g_ values of DnaGΔZBD oligomeric states, calculated using the computer program CRYSOL.^[^ ^31^ ^]^

**Figure 4:**
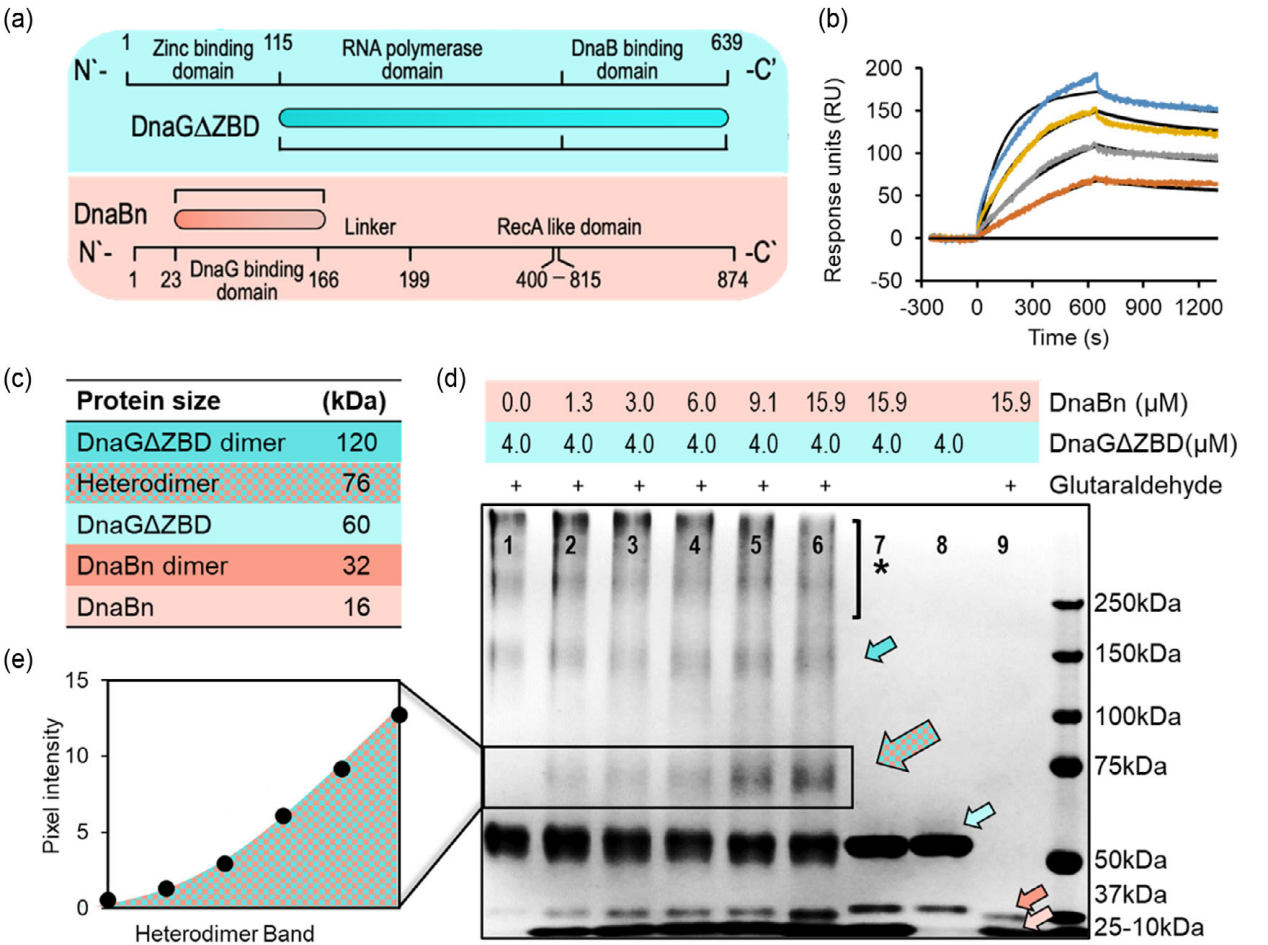
Interactions between primase–helicase domains (DnaGΔZBD–DnaBn) of *M. tuberculosis*. a) Schematic representation of the structures of full‐length DnaG and DnaB proteins and the truncated variants used in the current study (DnaG, turquoise, and DnaB, orange). b) SPR analysis of the interaction of soluble DnaGΔZBD with immobilized DnaBn. The response is plotted as a function of soluble DnaGΔZBD concentration, and steady‐state analysis revealed *K*_D_ = 0.21 ± 0.08 μM. c) Molecular weights of the expected protein complexes. Heterodimer refers to DnaGΔZBD–DnaBn complex. d) SDS‐PAGE analysis of cross‐linked DnaGΔZBD and DnaBn, at a constant glutaraldehyde concentration of 0.045%. The cyan arrow points to 60‐kDa DnaGΔZBD monomers; the blue arrow to 120‐kDa DnaGΔZBD dimers; and the orange and light‐orange arrows to 32‐kDa DnaBn dimers and 16‐kDa DnaBn monomers, respectively. The mosaic arrow indicates the 76‐kDa DnaBn–DnaGΔZBD heterodimer. The asterisk indicates the high‐molecular‐weight aggregates. e) Quantification of the pixel intensity of the 76‐kDa DnaBn–DnaGΔZBD heterodimer bands plotted as a function of DnaBn concentration.

Before conducting SAXS analysis, we determined the values of the radius of gyration (*R*_g_) for the monomer (24 Å) and the dimer (27 Å) of T7 primase using the software CRYSOL^[^ ^31^ ^]^ and the crystal structure of T7 primase (PDB ID: 1NUI^[^ ^29^ ^]^). The SAXS analysis indicated that upon the addition of 4‐mer RNA fragments (5′‐ACCC‐3′), the *R*_g_ values of the T7 primase reached the theoretical values of the dimer as the concentration of RNA increased (Figure 2b‐1). Furthermore, the distribution of pairwise distances within the protein, represented by *P*(*r*), revealed the protein size and phase shift with the addition of RNA (**Figure** 2b‐**3**). Importantly, in the absence of ATP/CTP, RNA, and Mg^2+^, T7 primase is a stable monomer, as the *R*_g_ values did not change with the increase of protein concentration (Figure 2b‐4).

**Figure 5:**
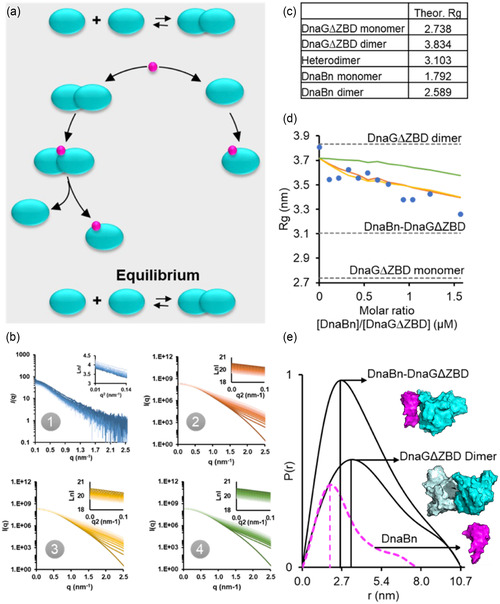
Low‐resolution SAXS model for disruption of DnaG dimers by DnaB. a) Schematic model for disruption of DnaG dimers by DnaB. b) SAXS profiles and the corresponding Guinier plots. Experimental data (blue, raw data are presented in Figure S3, Supporting Information). Simulation of the disruption of a DnaG dimer by a DnaBn monomer (orange). Simulation of the direct binding of a DnaBn monomer to a DnaG monomer (yellow). Simulation of noninteracting species (green). c) Theoretical *R*_g_ values for the DnaBn helicase, calculated using the crystal structure of the N‐terminal domain of DnaB helicase from *M. tuberculosis* (PDBID: 2R5U)^[^ ^13^ ^]^ and of DnaGΔZBD primase, calculated using the high‐resolution structure prediction I‐TASSER suite^[^ ^34^ ^]^ and the computer program CRYSOL.^[^ ^31^ ^]^ d) Simulated and experimentally determined *R*_g_ values of DnaBn helicase and DnaGΔZBD primase mixed in solution at different molar ratios, based on the determined *K*_D_ value and mass action law. The dashed lines represent the theoretical *R*_g_ values of the DnaGΔZBD monomer or homodimer or the DnaBn–DnaGΔZBD heterodimer in a solution, while the blue dots represent the *R*_g_ values of the interacting proteins in a solution, as obtained by SAXS. e) Experimentally determined distance distribution function *P*(*r*) of DnaBn and DnaGΔZBD species. Protein structure figures were created using the visualization software PyMol (https://pymol.org/). Theoretical SAXS profiles were generated using CRYSOL based on 3D atomic models of the oligomeric states. Experimental data were directly compared with theoretical curves to identify the molecular species present at each stage of the dissociation assay.

Protein models, reconstructed from SAXS measurements to visualize their overall shape, fit well with the crystal structure of a dimer (Figure 2b‐5). To reinforce the hypothesis that T7 primase forms dimers, we utilized two cross‐linking methods, namely ferrous‐catalyzed selective cross‐linking (Figure 2c) and glutaraldehyde cross‐linking (Figure 2d).

While glutaraldehyde cross‐linking is inherently a highly nonspecific process that involves most of the lysine residues distributed on the protein surface,^[^ ^32^ ^]^ ferrous‐catalyzed selective cross‐linking specifically targets the tyrosine residue pairs located on the monomer interaction surface.^[^ ^33^ ^]^ The resulting cross‐linked products were thoroughly analyzed using size exclusion chromatography (SEC) and matrix‐assisted laser desorption/ionization time‐of‐flight mass spectrometry (MALDI‐TOF, Figure 2c,d). Our analyses employing sodium dodecyl sulfate polyacrylamide gel electrophoresis (SDS‐PAGE) and SEC supported the formation of dimeric species of T7 primase, along with a minor population forming high‐molecular‐weight oligomers, predominantly trimers (Figure 2c,d). The MALDI‐TOF molecular weight analysis provided additional validation for these observations (Figure 2c,d). Collectively, these results align with and support the crystal structure of T7 primase as a homodimer, wherein the RPD of one monomer interfaces with the ZBD of the second monomer^[^ ^29^ ^]^ (Figure 2b‐5).

The study into the homodimer formation for T7 primase was expanded to *M. tuberculosis* DnaG. Surface plasmon resonance (SPR) analysis of the full DnaG shows preferential binding of DnaG to DnaG immobilized on an SPR chip (*K*_D_ = 2.0 ± 0.3 μM), which indicates dimerization (Figure 3a). The binding profile did not follow a pure 1:1 binding fit, probably due to the heterogeneity of the oligomeric states of the recombinant protein. The dimerization of DnaG was further validated by using glutaraldehyde cross‐linking on a truncated, more stable version of *M. tuberculosis* DnaG without the ZBD (DnaGΔZBD, amino acids 115‐639, **Figure** **4**a). For details on overexpression‐purification see Section 4. SDS‐PAGE analysis revealed the emergence of a high‐molecular‐weight protein species equivalent to the size of a DnaG dimer (≈120 kDa, Figure 3b). At the lowest concentration of 0.011% glutaraldehyde, the dominant band size corresponds to a monomer (≈60 kDa). More dimers were formed with increasing glutaraldehyde concentration—an apparent shift reaching an ≈1.5 monomer:1 dimer ratio was observed at 0.25% glutaraldehyde concentration (Figure 3b). The high‐molecular‐weight species that appear at the interface of the stacking and the running gel can be attributed to the nonspecific aggregate formation. The cross‐linking results were confirmed by analyzing the *R*_g_ values obtained from SAXS analysis (Figure 3c). The structure of DnaGΔZBD was predicted using the I‐TASSER suite^[^ ^34^ ^]^ and the computer program CRYSOL^[^ ^31^ ^]^ (Figure 3d). The *R*_g_ values of the species in a solution approached the theoretical values of a DnaGΔZBD dimer with the increase of DnaGΔZBD concentration (Figure 3c) and supported dimerization of *M. tuberculosis* DnaG, similarly to the T7 and other bacterial primases.

### 2.2 Physical Interactions between Helicase and Primase of *M. tuberculosis* Are Characterized by SPR and Cross‐Linking

When using a purified protein domain of DnaB for binding experiments instead of the full‐length protein enhances solubility, simplifies experimental conditions, and provides clearer insights into the molecular mechanisms of interactions. DnaG‐binding domain of DnaB (DnaBn, amino acids 23‐166, Figure 4a) was overexpressed and purified (see Section 4), and its physical interactions with DnaGΔZBD were followed using SPR analysis. The helicase domain was immobilized and subjected to increasing amounts of primase. Following each binding event, a wash step was conducted to remove unbound or loosely bound proteins from the sensor surface, ensuring that only specific interactions between DnaBn and DnaGΔZBD are measured. A steady‐state model fitting to averaged response units (RU) from SPR facilitated the determination of the *K*_D_ binding value (0.21 ± 0.08 μM, as shown in Figure 4b).

We found an additional linker of 35 amino acids, extending the modified DnaB protein and exposing the protein–protein interaction surface, making it more accessible to DnaGΔ ZBD when bound to the chip. The linker also provides two additional connecting points to the chip via Arg 169 and Arg 170, thus increasing the variety of available connecting positions for DnaGΔ ZBD interaction.

Determining the type of complex formed between the interacting domains of helicase and primase from the SPR signal is a challenging task despite the stability of the complex. Therefore, we used a cross‐linking assay to clarify the binding of helicase and primase to form a complex (Figure 4c). Utilizing glutaraldehyde to link the two protein domains chemically, we observed the formation of a heterodimer (DnaBn–DnaGΔZBD) as evidenced by the 76‐kDa band on the SDS‐PAGE. Figure 4d, indicated by mosaic arrow) with 0.045% glutaraldehyde and a two‐ to threefold excess of helicase over primase. Even with a substantial molar excess of helicase, the enhanced intensity of the gel bands suggests weak binding interactions between the protein domains. Notably, the presence of a 120‐kDa band in the sample containing only primase plus cross‐linking agent (Figure 4d, lane 1, marked with a cyan arrow) indicates the formation of a DnaGΔZBD dimer in the solution. The smearing in the region of high‐molecular weight indicates the presence of large protein species resulting from nonspecific cross‐linking, a common side effect of glutaraldehyde (Figure 4d, marked with an asterisk).

### 2.3 SAXS Dissociation Assay Indicates the Evolution of Binding between Helicase and Primase and the Transition of DnaG from Dimers into Monomers

X‐ray scattering by macromolecules in a solution is affected by factors such as molecular weight and electron density. The intensity of the scattering is directly proportional to the electron count of the molecule. Larger proteins with more atoms scatter more X‐rays, resulting in higher intensity. This allows us to track structural interactions between binding species in a solution using the SAXS reconstitution assay, a method specifically tailored to detect weak protein–protein interactions with dissociation constants (*K*_D_) in the millimolar range.^[^ ^21^, ^22^, ^23^ ^]^ Our SAXS dissociation assay was inspired by this reconstitution assay and the observation that a high‐molecular‐weight form of DnaG could be identified before the emergence of a lower‐weight species caused by dimer disruption following helicase binding to primase dimers. Based on our experimental data, we propose two models for the interaction between DnaB helicase and DnaG primase (**Figure** **5**a). The first model suggests a direct interaction between the DnaBn and DnaGΔZBD monomers, while the second model suggests the formation of a DnaG dimer, which is disrupted by DnaB binding, leading to the formation of a DnaB–DnaG heteromer. Based on these models, we generated the respective theoretical X‐ray scattering profiles (Figure 5b2–3) and calculated their theoretical *R*_g_ values (Figure 5c). Subsequently, we performed SAXS analysis on samples with a constant concentration of DnaGΔZBD and gradually increasing amounts of DnaBn. The experimental *K*_D_ values for DnaBn–DnaGΔZBD and DnaGΔZBD–DnaGΔZBD interactions (0.21 and 2 μM, respectively) were used for the data analysis. We observed a significant decrease in the *R*_g_ values (Figure 5d, blue circles) compared to the hypothetical reaction model where the two protein components do not interact (Figure 5e), and this correlated with the corresponding change in the *R*_g_ values obtained by SAXS (Figure 4d). The distance distribution function (*P*(*r*)), which illustrates the pairwise size distribution of the protein species assessed, shows a shift toward compactness upon the addition of DnaBn to DnaGΔZBD (Figure 5e).

It is important to note that SAXS analysis provides low‐resolution structural data that reveal overall molecular dimensions and solution conformations. Therefore, the SAXS‐derived models we present (Figure 5) reflect averaged solution structures rather than precise atomic arrangements. The previously reported high‐resolution crystal structure of the helicase C‐terminal domain bound to primase^[^ ^14^, ^16^ ^]^ demonstrates a specific architecture in which two helicase N‐terminal domains dimerize, allowing for the binding of a single primase C‐terminal domain. Although our SAXS‐derived structures do not explicitly capture atomic‐level detail, their overall shapes and dimensions are consistent with this configuration. Consequently, the structural models proposed here may indeed represent the biologically relevant architecture identified in these high‐resolution studies, though at a lower resolution.

## 3 Conclusion

The DnaB helicase of *M. tuberculosis* is believed to be involved in numerous protein–protein interactions, including those with DnaG primase, thus playing a crucial role in regulating DNA replication.^[^ ^27^ ^]^ This study indicates that DnaG forms dimers in solution, presumably the active form of primase, and that the binding of DnaB to DnaG forms heterodimers that destabilize the DnaG dimers. This can lead to a switch between two states: one, fast, coordinated lagging‐strand and leading‐strand synthesis DNA synthesis, and two, slow, where primase halts the DNA replisome and synthesizes RNA primer on the lagging DNA strand.^[^ ^35^ ^]^ This regulatory switching mechanism may apply to other bacterial DNA replication systems. This hypothesis was tested using triple‐resonance‐based NMR assignments under varying conditions.^[^ ^30^ ^]^ There, we observed spectral changes, confirming that nucleotide binding and Mg^2^^+^ induce a transition to a higher‐order species. Complementary SAXS and cross‐linking experiments further confirmed dimerization. We also adopted a similar experimental setup to evaluate this effect in the context of *M. tuberculosis* DnaG and to reinforce the generalizability of dimerization as a conserved structural feature among bacterial primases.

After confirming the presence of dimers of primase in solution, we utilized a SAXS dissociation assay, which is a rapid and cost‐effective method for monitoring the disruption of oligomers in solution. We confirmed that the contribution of a dimer of DnaGΔZBD (120 kDa) to the SAXS signal is significantly higher than that of the monomer (60 kDa), as the dimer has twice the amount of electron scatterers compared to the monomer. The contribution of DnaBn (16 kDa) to the SAXS signal is minor, even compared to the monomer of DnaGΔZBD. However, adding DnaBn could significantly alter the scattering pattern of a sample containing DnaGΔZBD if there is a notable change in particle size, like the disruption of a dimer of DnaGΔZBD. We combined this method with other biochemical tools, such as cross‐linking and SPR, that support the hypothesis that DnaGΔZBD dimers become unstable when DnaBn binds to them. While effective for isolating interaction regions, the use of truncated domains may not replicate the native hexameric assembly of DnaB or the full‐length monomeric DnaG. This limitation emphasizes the need for future studies with full‐length proteins under physiological conditions.

Furthermore, our SAXS data confirm the existence of heteromeric complexes and provide insights into their overall solution conformations. However, it is important to note that these data are inherently low‐resolution. As a result they may reflect the arrangement of helicase and primase domains previously revealed by high‐resolution studies.^[^ ^14^, ^16^ ^]^ To conclusively validate and refine our proposed solution structures, future studies employing higher‐resolution methods such as cryo‐electron microscopy or X‐ray crystallography are recommended.

It is important to note that our findings demonstrating stable dimer formation by DnaGΔZBD should be interpreted in the context of existing literature on primase oligomerization. Unlike the full‐length DnaG primase, the truncated DnaGΔZBD variant used in our study inherently favors stable dimer formation. Previous studies on the isolated C‐terminal HBD of primase have similarly demonstrated stable dimerization in solution.^[^ ^15^, ^16^ ^]^

However, in biological contexts, bacteriophage T7 primase does not form stable independent dimers in vivo but rather participates in functionally essential dimeric interactions within the gp4 helicase hexamer during primer synthesis. Similarly, *E. coli* DnaG primase predominantly operates as a monomer during RNA primer synthesis, but it transiently interacts with the DnaB helicase to form the primosome and with DNA polymerase III holoenzyme via the τ (DnaX) subunit. Therefore, while our structural findings with DnaGΔZBD provide valuable insights into possible oligomerization interfaces and interactions, we emphasize that the functional oligomerization state of full‐length DnaG in vivo remains predominantly monomeric, regulated by dynamic protein–protein interactions at the replication fork.

Understanding the nature of interactions between proteins in the replisome is crucial for developing novel small molecules that disrupt the interactions between DnaB and DnaG or prevent the disruption of DnaG dimers by DnaB, thereby halting DNA replication and inhibiting bacterial proliferation.

## 4 Experimental Section

4.1

4.1.1

##### Materials

Chemicals used for biochemical assays were of molecular‐biology grade and were purchased from Sigma‐Aldrich. Ribonucleotides (ATP, CTP, GTP, and UTP) were purchased from New England Biolabs (NEB). [α–^32^P]ATP (3000 Ci mmol^−1^) and [γ–^32^P]ATP (3000 Ci mmol^−1^) were purchased from Perkin Elmer.

##### T7 Primase Expression and Purification

The primase domain of gene 4 protein (gp4) from bacteriophage T7 (residues 1–271) was overexpressed and purified as previously described.^[^ ^30^, ^36^ ^]^

##### *M. tuberculosis* DnaG and DnaGΔZBD

The DNA sequences of DnaG and DnaGΔZBD (amino acids 115‐639) were cloned into pET28 vectors and transformed into *E. coli* BL21 DE3. The cells were grown in LB medium with 60 mg mL^−1^ kanamycin to an OD600 of 0.6, induced with 0.5 mM IPTG, and incubated for 4 h at 37 °C and 150 rpm. After induction, cells were collected by centrifugation, resuspended in Buffer G1 (50 mM Tris–HCl pH 7.5, 150 mM NaCl, 1 mM DTT, 10 mM imidazole), and lysed using a high‐pressure homogenizer and sonication. The lysate was clarified by centrifugation, and the supernatant was loaded onto a Ni‐NTA column. The column was washed with Buffer G1 containing 10 mM imidazole, and the protein was eluted with Buffer G1 using an imidazole gradient up to 500 mM. Protein‐containing fractions were combined, EDTA was added to 1 mM, and proteins were concentrated by ammonium sulfate precipitation and ultracentrifugation. The protein precipitate was dissolved in Buffer G2 (50 mM Tris–HCl pH 7.5, 1 mM EDTA, 1 mM DTT, 500 mM NaCl, 10% glycerol), loaded onto a Sepharose S‐200 column, and eluted with Buffer G2. Fractions containing the protein were combined, subjected to another ammonium sulfate precipitation, dissolved in Buffer G2, and dialyzed against storage buffer (50 mM Tris–HCl pH 7.5, 1 mM EDTA, 1 mM DTT, 500 mM NaCl, 50% glycerol). Protein yield was 2 mg L^−1^ of culture. The two proteins were stored at −20 °C. DnaGΔZBD is considered more stable, as it can be overexpressed in larger amounts and can reach sub‐millimolar concentrations in solution without precipitation, compared to full‐length DnaG.

##### *M. tuberculosis* DnaBn

To express *M. tuberculosis* DnaBn (amino acids 23‐166) in *E. coli* BL21 DE3, the DNA sequence was amplified from pET28‐DnaB, which incorporated NdeI and HindIII recognition sites, by using specific primers. The amplified DNA and pET28 plasmid were digested with NdeI and HindIII, ligated with T4 DNA ligase, and transformed into *E. coli* BL21 DE3 for protein expression.

For protein production, cells were grown in LB medium with 2% glucose and 5% glycerol at 37 °C and 175 rpm. At OD_600_ = 0.6, expression was induced with 0.5 mM IPTG, and cells were incubated at 18 °C and 150 rpm for 20 h. Cells were harvested by centrifugation, resuspended in Buffer T1 (40 mM Tris–HCl pH 8, 400 mM NaCl, 1 mM DTT, 5 mM imidazole), and lysed by sonication. The lysate was clarified by ultracentrifugation, and the supernatant was loaded onto a Ni‐NTA agarose gravity column. The column was washed with Buffer T1 containing 5 mM imidazole, and the protein was eluted in steps with Buffer T containing 50 and 500 mM imidazole.

Protein‐containing fractions were identified by SDS‐PAGE, pooled and concentrated by ammonium sulfate precipitation, and followed by ultracentrifugation. The protein precipitate was dissolved in buffer T2 (40 mM Tris–HCl pH 8, 200 mM NaCl, 2 mM DTT, 0.1 mM EDTA) and purified on a Superdex‐100 column. Protein concentration was determined using a NanoDrop spectrophotometer (Thermo) at 280 nm, yielding 1.65 mg L^−1^ of protein. The protein was stored in a storage buffer (50 mM Tris–HCl pH 7.5, 500 mM NaCl, 1 mM DTT, 1 mM EDTA, 50% glycerol) at −20 °C.

##### 2D NMR Measurements and Analysis of T7 Primase

[^1^H, ^15^N]TROSY HSQC spectra of ^15^N‐deuterated T7 DNA primase in three states—free, bound to ATP/CTP, or bound to ATP/CTP, and Mg(II)—were recorded at 25 °C on a Bruker DMX 800 MHz spectrometer equipped with TXI cryoprobes with Z gradient. Data were processed and analyzed as described before.^[^ ^37^ ^]^ The NMR assignments used for interpreting chemical shift perturbations have been previously reported.^[^ ^30^ ^]^ In that study, backbone resonances were assigned using standard TROSY‐based triple resonance experiments (HNCA, HNCOCA, HNCO, HNCACO, and HNCACB), allowing us to confidently map chemical shift changes to specific amino acid residues. We obtained ≈70% backbone resonance assignments for a sample of T7 primase fragment (residues 1‐271). The assignment is presented in Figure S1a, Supporting Information, and the structural changes observed in T7 primase upon nucleotide and magnesium addition are presented in Figure S1b–c, Supporting Information.

##### SAXS

T7 primase was analyzed in a SAXS buffer containing 20 mM Tris–HCl pH 7.5 and 1 mM DTT at a concentration range of 0.5–8 mg ml^−1^. In the case of the T7 primase and RNA primer complex, a constant concentration of 1 mg ml^−1^ of T7 primase was mixed with increasing concentrations of short RNA fragments (5′‐ACCC‐3′) at an RNA: protein molar ratio ranging from 1 to 10. Measurements were performed in beamline BM29 at the European Synchrotron Light Source (ESRF, Grenoble). The X‐ray wavelength was 1.5 Å, and the temperature was 4 °C. The detector was Pilatus 1 M, and the sample‐to‐detector distance was set at 2.86 m, with a scattering vector (*q*) range of 0.0025–0.5 Å^−1^. At a scattering angle of 2*θ*, the magnitude of the scattering vector (*q*) is defined as

$$q=\frac{4\pi \sin\theta }{\lambda }$$

The experimental SAXS data for all samples were linear in the low *q* Guinier region. The radii of gyration (*R*_g_) were derived from data in the *qR*_g_ < 1 region by using the Guinier approximation

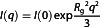




We analyzed the small‐angle region (0.012 < *q* < 0.08 Å^−1^) of the scattering profiles using the Guinier approximation embedded in the ATSAS suit.^[^ ^31^ ^]^ The scattering curve reflects structural characteristics in reciprocal space. Fourier transformation translated scattering profiles into real space, distance distribution function *P*(*r*), using the software GNOM^[^ ^38^ ^]^ and in‐house scripts^[^ ^39^ ^]^ to obtain a reliable quantification of the longest cord of the particle (*D*_max_) deduced from the *P*(*r*). The *R*_g_ of monomeric/dimeric proteins used in this study extracted from SAXS data using the computer program CRYSOL^[^ ^31^ ^]^ and the crystal structure of DnaB of *M. tuberculosis* (PDBID: 2R5U)^[^ ^13^ ^]^ and the DnaGΔZBD obtained by using I‐TASSER.^[^ ^34^ ^]^ The overall three‐dimensional ab initio models complexes were restored using DAMMIN.^[^ ^40^ ^]^ For all samples, 10 low‐resolution models were averaged using the program DAMAVER^[^ ^41^ ^]^ to yield an averaged model representing the general structural features of each reconstruction.

The SAXS dissociation assay employed in this study was inspired by the SAXS reconstitution approach described previously,^[^ ^21^ ^]^ which is highly sensitive for detecting transient or weak protein–protein interactions in solution. In this work, we adapted the methodology to specifically monitor the dissociation of DnaG primase oligomers upon binding of DnaB helicase. Initial SAXS measurements revealed a high‐molecular‐weight form of DnaGΔZBD, consistent with dimerization, as indicated by the experimentally derived radius of gyration (*R*_g_). By gradually increasing the concentration of DnaBn in a solution containing constant DnaGΔZBD, dissociation events could be monitored. Elevation of DnaBn concentration resulted in a gradual decrease in *R*_g_, corresponding to the disruption of the dimer and the formation of lower‐order species, most likely DnaB–DnaG heteromers.

To interpret the experimental SAXS data, theoretical SAXS profiles were generated using CRYSOL software,^[^ ^31^ ^]^ which calculates theoretical scattering curves based on atomic coordinates obtained from atomic structures. For our analysis, we used available crystal structures from the Protein Data Bank (PDB ID: 1NUI for T7 primase and 2R5U for the *M. tuberculosis* helicase domain) or models generated by structure prediction software (I‐TASSER for DnaGΔZBD.^[^ ^34^ ^]^ These theoretical SAXS profiles served as benchmarks for direct comparison with experimental SAXS data.

##### Fe(II)‐Catalyzed Crosslinking of T7 DNA Primase

T7 primase (15 μM) was incubated with 1 mM ATP and 0.5 mM Fe(II)‐chloride on ice for 5 min. An additional volume of 10 mM Tris–HCl pH 7.5, 0.15% H_2_O_2_, 5 mM ascorbic acid, and 1 mM ferrous chloride was added, and the reaction was incubated at room temperature for 45 min. The reaction was quenched with 5 mM EDTA, concentrated to ~200 μM, and buffer‐exchanged to 50 mM Tris–HCl pH 7.5, 1 mM EDTA, 1 mM DTT, and 100 mM NaCl. The sample was loaded onto a Superdex 200 column and eluted with the same buffer.

##### Crosslinking with Glutaraldehyde (T7 DNA Primase)

Glutaraldehyde (0.01%) was added to a mixture of 40 mM Tris–HCl pH 7.5, 10 mM DTT, 50 mM potassium glutamate, 0.2 mM ATP, 0.2 mM CTP, 1 mM Mg, and 17 μM T7 DNA primase. The sample was incubated at room temperature for 30 min, and then quenched with 80 mM Tris–HCl pH 8. The sample was concentrated to ≈300 μM, buffer‐exchanged, and eluted on a Superdex 200 column with 50 mM Tris–HCl pH 7.5, 1 mM EDTA, 1 mM DTT, and 100 mM NaCl.

##### Crosslinking *M. tuberculosis* DnaGΔZBD and DnaBn with Glutaraldehyde

For DnaGΔZBD, 4 μM of the protein was incubated with glutaraldehyde (0.011–0.25%) in 40 mM HEPES pH 7.5, 150 mM NaCl, 1 mM DTT, 0.1 mM EDTA, and 0.5% DMSO for 30 min at 25 °C. For interactions with DnaBn, 4 μM DnaGΔZBD was incubated with DnaBn (0–15.9 μM) in 0.045% glutaraldehyde, 40 mM HEPES (pH 7.5), 150 mM NaCl, 10 mM MgCl_2_, 0.1 mM EDTA, and 1 mM DTT for 25 min at 25 °C. Reactions were quenched with 1 M Tris–HCl pH 8 and incubated for 10 min at room temperature. Samples were analyzed by SDS‐PAGE and Coomassie blue staining. The pixel intensity of gel bands was quantified using ImageJ.^[^ ^42^ ^]^

Although glutaraldehyde cross‐linking can yield nonspecific higher‐order aggregates, we verified the structural relevance of the main dimeric species using selective ferrous‐catalyzed cross‐linking, SAXS analyses, and MALDI‐TOF mass spectrometry. Together, these approaches confirm the biological relevance of the dimeric species identified.

##### SPR

SPR experiments were performed on a ProteOn XPR36 (Bio‐Rad Laboratories), using a ProteOn GLC sensor chip (Bio‐Rad Laboratories) with amine coupling by sulfo‐NHS (0.1 M N‐ 27 hydroxysuccinimide) and EDC (0.4 M 1‐ethyl‐3‐(3dimethylaminopropyl)‐carbodiimide). Proteins of interest or bovine serum albumin (BSA) (3 μg, negative control) were covalently attached to the surface of the chip in 10 mM sodium acetate buffer pH 4.0. Unbound esters were deactivated with 1 M ethanolamine HCl at pH 8.5. To determine the *K*_D_ for the binding reaction between the full‐length DnaG monomers, 7.2 μg of the protein was immobilized on the surface of the chip, and different concentrations of soluble DnaG (0, 1.06, 2.125, 4.25, 8.5 and 17 μM) were allowed to flow over the chip at a flow rate of 60 μL min^−1^ for 1600 s. The injection volume was 409 μL

To determine the *K*_D_ for the binding reaction of DnaGΔZBD with DnaBn, 2.4 μg DnaBn was immobilized on the surface of the chip, and different concentrations of soluble DnaGΔZBD (0, 37.5, 75, 150, 300, and 600 nM) were allowed to flow over the chip at a flow rate of 30 μL min^−1^ for 1300 s. The injection volume was 150 μL. Binding was determined at 25 °C with 40 mM HEPES, pH 7.5, 200 mM NaCl, 0.1 mM EDTA, and 1 mM DTT as running buffer. The interactions obtained were normalized to the RU values of the BSA‐immobilized channel. Binding affinity values were determined using binding equilibrium and two‐step kinetic equations. These steps were repeated three times to establish the *K*_D_ value for each protein pair.

## Conflict of Interest

The authors declare no conflict of interest.

## Author Contributions

**Dayan A**: experimentation, data analysis, writing. **Ilic S**: experimentation, data analysis, writing. **Akabayov B**: supervision, conceptualization, experimentation, data analysis, visualization, writing.

## Supporting information

Supplementary Material

## Data Availability

The data that support the findings of this study are available from the corresponding author upon reasonable request.
