# Supplementary material for: Characterization of DnaB–DnaG Interaction in M. tuberculosis Using Small‐Angle X‐ray Scattering‐Based Dissociation Assay
Source: Chembiochem. 2025 Jul 1;26(14):e202500289. doi: 10.1002/cbic.202500289 (PMC12278352; doi:10.1002/cbic.202500289)
Supplement: Supplementary file 1 — Supplementary Material [file CBIC-26-e202500289-s001.pdf]

SUPPORTING INFORMATION

DECODING DNAB-DNAG COMPLEX FORMATION IN *M. TUBERCULOSIS* BY USING A SMALL-ANGLE X-RAY SCATTERING (SAXS) DISSOCIATION ASSAY

Adi Dayan, Stefan Ilic, Barak Akabayov
From the Department of Chemistry and Data Science Research Center, Ben-Gurion University of the Negev, Beer-Sheva 8410501, Israel.

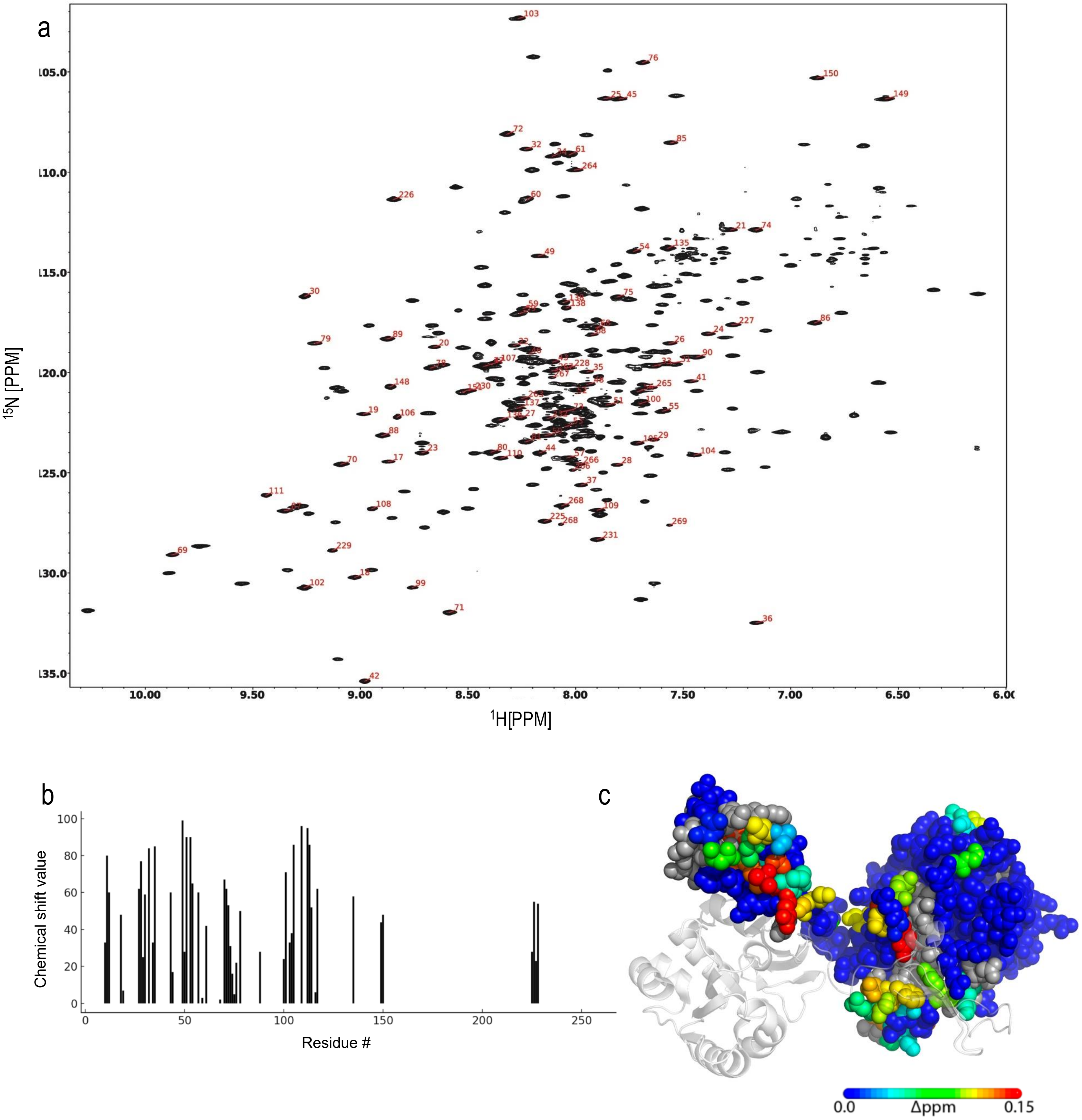

Fig. S1: Observation of DNA primase dimerization. (a) Assignments of the observed resonances were derived from published NMR assignments of T7 primase (Ilic et al., 2016). (b) Quantification of chemical shift perturbation. Plotted are the chemical shift perturbation values against the amino acid sequence of T7 primase, showing the residues with the largest perturbations. (c) The chemical shift perturbation values were mapped onto the crystal structure of T7 primase (PDB ID: 1NUI), and the residues were colored according to the magnitude of the shift.

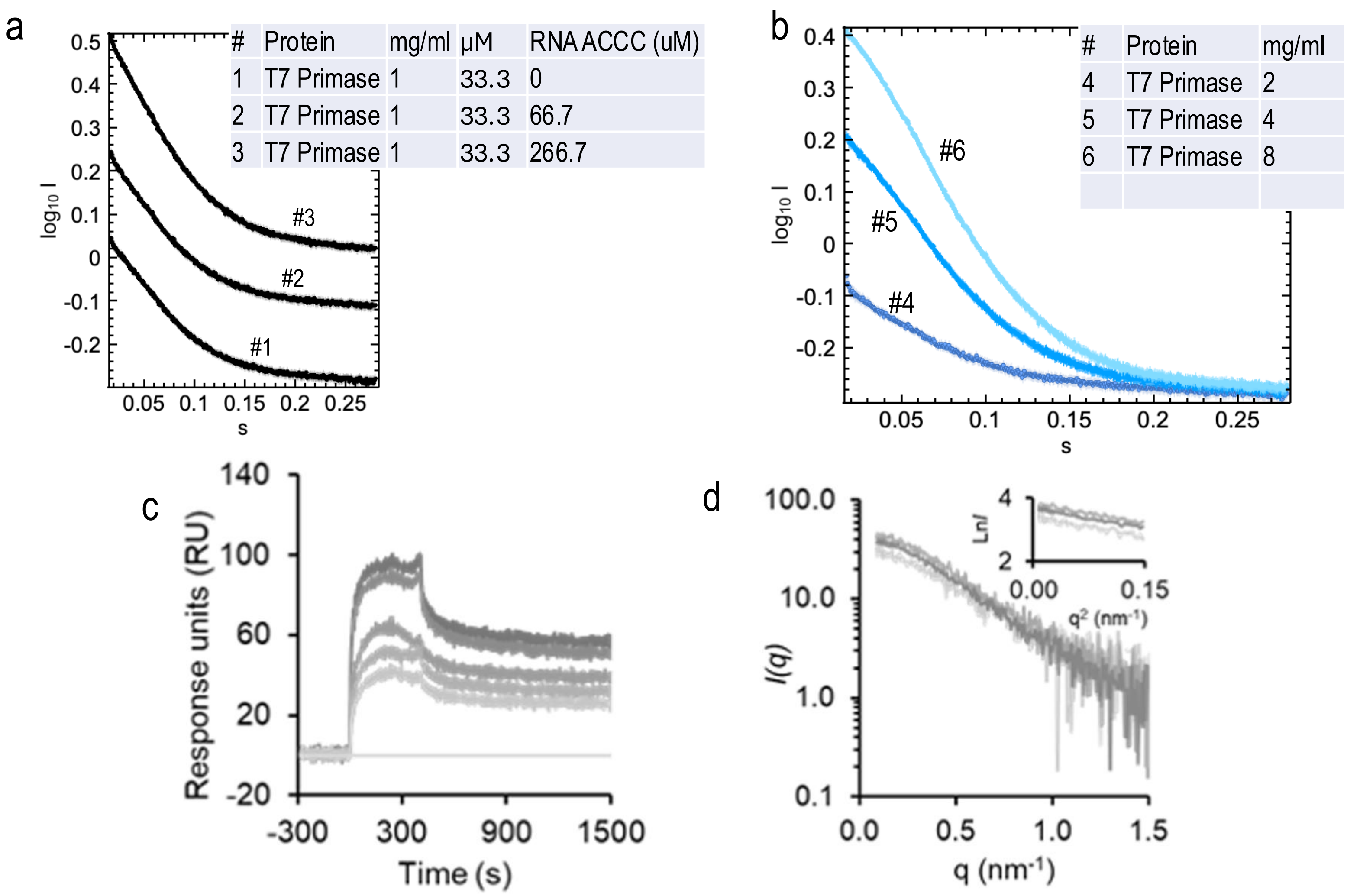

#	Protein	mg/ml	µM	RNA ACCC (uM)
1	T7 Primase	1	33.3	0
2	T7 Primase	1	33.3	66.7
3	T7 Primase	1	33.3	266.7

#	Protein	mg/ml
4	T7 Primase	2
5	T7 Primase	4
6	T7 Primase	8

Fig. S2: Observation of DNA primase dimerization (cont'd). (a) SAXS profiles of T7 primase (50 µM) in the presence of increasing amounts of 4-mer RNA primers (5'-ACCC-3'). A summary of the sample components and their quantities is presented in the table (inset). (b) SAXS profiles of free T7 primase in three concentrations. A summary of the sample components and their quantities is presented in the table (inset). (c) SPR analysis of soluble DnaGΔZBD binding to immobilized DnaGΔZBD on a GLC sensor chip using Amino coupling. (d) SAXS profiles of DnaGΔZBD at 5, 7, and 11 µM concentrations.

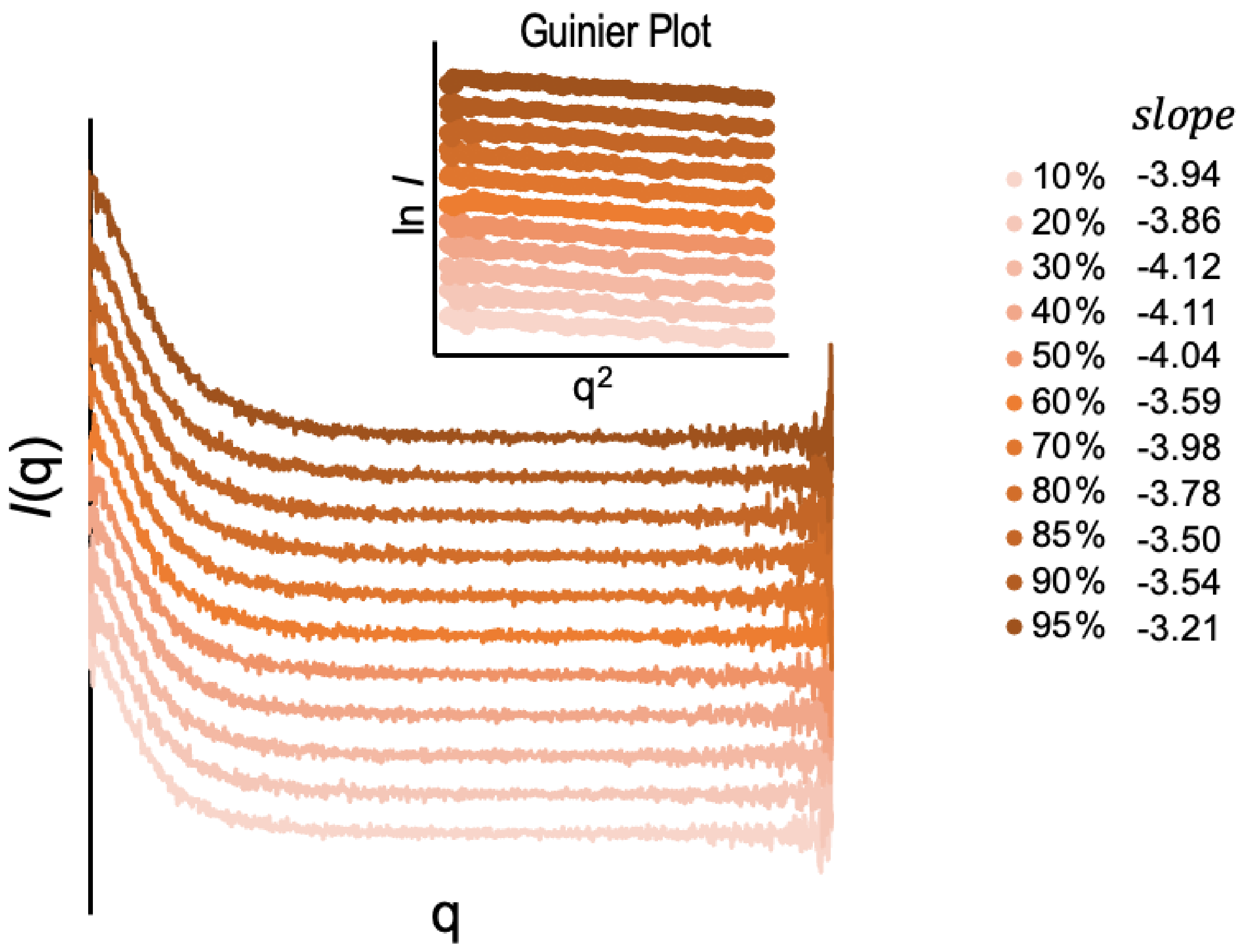

Fig. S3. SAXS profiles for DnaB titration on DnaGΔZBD. Inset: Guinier plots for spectra. The increasing I(0) values (from light to dark color) represent the spectra of a mix of DnaGΔZBD 7 μM and increasing amounts of DnaBn 0.6-19.0 μM.
